# Are the effects of air pollution on birth weight modified by infant sex and neighborhood socioeconomic deprivation? A multilevel analysis in Paris (France)

**DOI:** 10.1371/journal.pone.0247699

**Published:** 2021-04-15

**Authors:** Séverine Deguen, Wahida Kihal-Talantikite, Morgane Gilles, Arlette Danzon, Marion Carayol, Denis Zmirou-Navier

**Affiliations:** 1 School of Public Health (EHESP), DSET&GS, Rennes CEDEX, France; 2 Department of Social Epidemiology, Institut Pierre Louis d’Epidémiologie et de Santé Publique (UMRS 1136), Sorbonne Universités, UPMC Univ Paris 06, INSERM, Paris, France; 3 Laboratoire Image Ville Environnement, LIVE UMR 7362 CNRS, University of Strasbourg, Strasbourg, France; 4 Service de Protection Maternelle et Infantile, Direction des Familles et de la Petite Enfance, Mairie de Paris, Paris, France; 5 School of Medicine, Lorraine University, Nancy, France; 6 Inserm, Irset (Institut de recherche en santé, environnement et travail), UMR-S 1085, Rennes, France; Charite Universitatsmedizin Berlin, GERMANY

## Abstract

Adverse birth outcomes related to air pollution are well documented; however, few studies have accounted for infant sex. There is also scientific evidence that the neighborhood socioeconomic profile may modify this association even after adjusting for individual socioeconomic characteristics. The objective is to analyze the association between air pollution and birth weight by infant sex and neighborhood socioeconomic index. All birth weights (2008–2011) were geocoded at census block level. Each census block was assigned a socioeconomic deprivation level, as well as daily NO_2_ and PM_10_ concentrations. We performed a multilevel model with a multiple statistical test and sensible analysis using the spline function. Our findings suggest the existence of a differential association between air pollution and BW according to both neighborhood socioeconomic level and infant sex. However, due to multiple statistical tests and controlling the false discovery rate (FDR), all significant associations became either not statistically significant or borderline. Our findings reinforce the need for additional studies to investigate the role of the neighborhood socioeconomic which could differentially modify the air pollution effect.

## Introduction

There is strong scientific evidence of the adverse health effects of air pollution, including on birth outcomes such as low (<2500 g) and very low (<1500 g) birthweight, as well as on prematurity [1, 2]. Fetuses are considered highly vulnerable to environmental exposures including ambient air pollutants [1, 2]. More specifically, both low birthweight and preterm birth can be caused by maternal, fetal or placental factors, or indeed a combination of all three. For instance, air pollution may affect maternal health, which could in turn impair utero-placental and umbilical blood flow, transplacental glucose and oxygen transport: all consequences of poor maternal health are known to be major determinants of fetal growth [3, 4].

It is also well documented that certain windows in pregnancy predispose to higher risk for fetal development in relation to higher rates of cell proliferation and metabolic changes [1, 2]. The third trimester has been cited as a window at greater risk for reduction of birth weight related to air pollution, one advanced explanation being that birth weight gain is very important after the 28^th^ week of pregnancy [1, 2]. Chen *et al*. found that an increase of 10 μg/m^3^ of PM_10_ during the third trimester reduced birth weight by 11g (95% CI, 2.3–19.8). However, different critical trimesters of exposure have been reported in the literature, according to the birth outcomes of interest and/or the pollutants considered. Xu et al. [5] found significant associations between term low birth weight (TLBW) and exposure to PM_10_ during the first trimester as well as the second trimester, while Ha et al. [6] showed a significant increase to TLBW risk with exposure to PM_2.5_ during the second trimester only.

While these studies have enhanced our understanding of the risks caused by air pollution to fetuses, caution should be exercised in interpreting their findings where the data analysis has ignored the male disadvantage hypothesis. Despite the weight advantage (infant birth weight is on average higher among males than female infants), perinatal mortality is more frequent among newborn males. Also, in the low or very low birth weight subgroup, the survival rate is lower among newborn males [7]. Growth of the male fetus is also slowed by extraneous factors such as smoking or exposure to air pollutants [8]. According to the Barker hypothesis, poor fetal growth is suspected to be a risk factor for adverse health later in life [9, 10]. To our knowledge, few studies have accounted for infant sex while examining the impact of air pollution on birth weight [7]. More precisely, the possible differential effects of air pollution on pregnancy outcomes have been revealed according to infant sex. Yet, the systematic review of 11 studies found some evidence of interaction between gender and exposure level [7]. For instance, Ghosh et al. [7], revealed that the risk of Low Birth Weight (LBW) would be higher in males with high air pollution exposure. However, underlying mechanisms for this infant sex difference are not well understood and demand further epidemiological and toxicological investigation.

Finally, the need to integrate both the socioeconomic context and environmental exposures into health research is well established, including when dealing with newborn health: pregnant women who live in socioeconomically deprived areas may be exposed to higher levels of pollution (differential exposure) and/or may be more vulnerable due to poor health conditions linked with socioeconomic deprivation (differential vulnerability). Moreover, there is substantial evidence for the adverse impacts of neighborhood socioeconomic deprivation on birth outcomes, even once individual socioeconomic characteristics have been taken into account [11, 12].

The majority of published studies have analyzed infant sex as a confounder, without considering that it could be an effect modifier of the association between birth outcomes and air pollution.

Indeed, it seems relevant to take into account both: *i)* the possible differentiate health impact of environmental exposure according to socioeconomic status and *ii)* the difference between newborn male and female infants in terms of weight at birth, for instance, or in terms of death rate at birth.

In the present study, the sex -specific effects of air pollution, taking socioeconomic status into account, were explored because of suspected differential vulnerability in utero due to air pollution exposure.

The main objective of our work is to analyze the association between birth weight by infant sex and air pollution or/and neighborhood socioeconomic index. First, we will analyze whether the air pollution effect on birth weight differs according to infant sex, in order to investigate the hypothesis that the air pollution effect could be higher among newborn male infants. Secondly, we will reiterate same analysis separately among male and female infants, taking neighborhood socioeconomic level into account; here, we will aim to investigate the hypothesis that the health effects of air pollution could be higher among people living in deprived neighborhoods, while taking infant sex into account.

To do this, we used data from medical certificates collected in the city of Paris, France, to investigate two main questions. Firstly (with a view to addressing the knowledge gap about infant sex differences) whether, in terms of the aforementioned associations between air pollution and birth weight, there is a difference between male and female infants. Secondly, whether these associations could vary in line with neighborhood socioeconomic characteristics estimated at a census block level.

## Material and methods

### Study area

The study area is the city of Paris, which has about 2,250,000 inhabitants. Paris is subdivided into 992 census blocks with a mean population of about 2,270 inhabitants and a mean area of 0.11 km^2^. A census block (named IRIS: *Ilots Regroupés pour l’Information Statistique* and defined by the National Institute of Statistics and Economic Studies—INSEE) corresponds to the smallest spatial unit whose aggregate data can be used on a routine basis.

### Study population

This study includes a population of newborns in Paris between 2008 and 2011. Individuals’ characteristics are available from the first medical certificate information registered by the Maternal and Child Care department of Paris (named PMI—*Protection Maternelle et Infantile*). This certificate is completed by parents and a health professional prior to leaving the maternity unit and then sent to the PMI for the department of residence. The data is recognized as having a high rate of completeness, covering about 93% of total births in Paris [13].

The medical certificate provides information about the newborn and his/her parents. This study includes the newborn characteristics: birth weight (in grams) as well as parent characteristics: maternal age (in years), occupational status of both parents (whether economically active or not), maternal level of education (primary, secondary, bac or higher education) and pregnancy characteristics: gestational age (in weeks), primiparous (yes or no), and maternity classification in terms of newborn and/or maternal health, (Class I: low risk pregnancy, Class II: moderate risk pregnancy or Class III: high risk pregnancy).

Ethical approval was granted by the French Data Protection Authority (CNIL). For reasons of confidentiality, and to comply with the ethical authorization accorded for this study (authorization number from the ethical institution: 914118, granted 18 November 2015), it was not possible to use the individual localization of the newborn. All postal addresses for maternal residency were geocoded at census block level. The number of cases was aggregated at census block level for statistical analysis; consent for study participation was thus not required. All experiments were carried out in accordance with relevant guidelines and regulations.

### Assessment of exposure to air pollution

To assess women’s cumulative exposure in the course of the pregnancy, we retrospectively estimated daily concentrations of pollutant at census block level, per trimester of pregnancy. Exposure to NO_2_ and PM_10_ was chosen because: *i)* many epidemiological studies, toxicological evidence and WHO reviews support the view that these pollutants have a causal relation with adverse birth outcome; *ii)* of the various air pollutants reported in the city of Paris, these are the two main pollutants; *iii)* for these two pollutants, the modeling approach performed by the local French association (AirParif), which monitors air quality in Paris, allows us to obtain accurate modeled estimates.

More precisely, daily concentrations of NO_2_ and PM_10_ were estimated for each census block by combining annual average concentrations modeled at census block level with daily variations of its index monitor (more details are provided in Deguen et al. 2015 and Kihal et al. 2016 [14, 15]).

Our data for this operation came from fixed monitoring stations (both background and traffic stations) located within the city of Paris, and from a deterministic model named ESMERALDA (www.esmeralda-web.fr) which integrates a range of input parameters: linear, surface and industrial point sources, as well as meteorological data [16]. The modeled data were obtained from AirParif air quality monitoring networks for the Ile-de-France (Parisian) region.

Next, a proxy of individual exposure per trimester of pregnancy was derived for each newborn, using date of birth, gestational age, and census block of residence.

### Socioeconomic level of neighborhood census blocks

The socioeconomic level of census blocks was assessed using a composite deprivation index which captures several socioeconomic aspects: family structure, household type, immigration status, employment, income, education and housing. Data for Paris are available from the National Institute of Statistics and Economic Studies (INSEE—2012). Principal component analysis was used to select those socioeconomic variables most correlated with the first principal component, then to calculate the socioeconomic deprivation value of each census block (further methodological details are provided in Lalloué et al., 2013 [17]). This deprivation index was categorized into five classes of census block, by quintile of distribution (from Class 1: least deprived to Class 5: most deprived census blocks). This socioeconomic index has been validated by previous ecological studies, which have demonstrated its ability to capture environment-related socio-spatial inequalities in France [13, 14].

### Statistical analysis

#### Outcomes

Three outcome variables were defined using the birth weight of the newborn. The Birth Weight (BW) was first considered as a continuous variable expressed in grams. Two additional outcomes were derived from the BW as binary variables: LBW defined as a weight below 2500 grams, and TLBW defined as a weight below 2500 grams at a gestational age > 36 weeks.

#### Confounders

Descriptive analysis revealed high rates of missing values for several individual socioeconomic variables: 42.4% (maternal) and 31.7% (paternal) occupational status data are missing, while about 29% of maternal level of education data is missing. In order to avoid imputation biases, we decided not to include these three variables in the analysis; this issue is addressed in the discussion section. Explanatory variables considered in the statistical analysis were: maternal age, gestational age, primiparous (or not), and maternity classification.

#### Models

Three different models were implemented: Models 0 and 1 consider individual data only, while Model 2 combines both individual and census block data. Model 0 corresponds to the adjusted referent model, including the following four explanatory variables: maternal age, gestational age, primiparous (or not), and maternity classification. In Model 1, individual exposure to air pollution for different periods of pregnancy was added. To do this, four windows of exposure were considered: trimester 1 (T1), trimester 2 (T2), and trimester 3 (T3), plus the entire pregnancy period as a whole. The three trimesters have been introduced simultaneously in the model. Model 2 includes the neighborhood socioeconomic index estimated at the census blocks level; more precisely, it includes two-level analysis in order to assess how much of the variability in instances of BW, LBW and TLBW respectively can be explained by individual level (Level 1) and census block level (Level 2) data. Multilevel modeling was justified by the design of the study in which individuals, i.e. the newborns (level 1), are nested within census blocks (level 2).

Regression coefficients (*β*) were estimated using both classical (Model 0 and Model 1) and multilevel (Model 2) linear regressions for associations between BW and explanatory variables, while classical (Model 0 and Model 1) and multilevel (Model 2) logistic regressions were used for LBW and TLBW (the odds ratios (OR) were produced with a 95% confidence interval).

Two different indicators were used to quantify between-census-block variations. Variations in BW were assessed using the intra-groups correlation coefficient (ICC). Median odds ratios (MORs) were computed to translate between-census-block variation in LBW and TLBW risks into an odds ratio scale. An MOR indicator of close to 1 means there was little variation between census blocks, whereas a high value indicates stronger between-census- block variation.

All analyses were stratified by infant sex, because the literature documents the existence of a “male disadvantage hypothesis” related to air pollution exposure [7]. In addition, interaction between air pollution and neighborhood socioeconomic level were analyzed in Model 2. All statistical analyses were conducted with alpha risk equal to 5%. Since we conducted multiple statistical tests, we controlled the False Discovery Rate (FDR) at α = 0.05, using the Benjamini & Hochberg method [18].

## Results

### Population description

A total of 115,112 newborns were recorded during the study period 2008–2011. After excluding births where BW or gestational age was unknown, and BWs of 500g or less (combined, amounting to 3.8% of total births), we counted 110,746 newborns. We also excluded newborns where the maternal residence address was missing (about 4.9%), because this precluded estimation of both exposure to air pollution and census block level socioeconomic deprivation. The final total of newborns included in the study is 105,346. Newborn characteristics are summarized in Table 1.

**Table 1:** Descriptive statistics for the population.

	GIRLS	BOYS
Newborn characteristics	Percentage	Percentage
Birth Weight (gr)*	3265 (σ = 456)	3392 (σ = 477)
Low Birth Weight	4.4%	3.2%
Term Low Birth Weight	2.5%	1.4%
**Parent characteristics**		
*Maternal age*		
<20 years	0.6%	0.6%
[20–25] years	6.4%	6.4%
[25–35] years	59.3%	59.7%
[35–40] years	25.9%	25.7%
≥ 40 years	7.8%	7.6%
*Maternal level of education*		
Primary	17.9%	18.1%
Secondary	12.5%	12.7%
High School	15.6%	15.3%
University	54.0%	53.9%
*Maternity Classification*		
Class I	28.0%	27.7%
Class II	42.8%	42.8%
Class III	29.2%	29.4%
Woman’s first pregnancy	50.9%	51.4%
Maternal occupational status	26.2%	26.2%
Paternal occupational status	20.3%	20.1%

* We gave the mean (standard deviation) of birth weight expressed in grams, as it is a quantitative variable.

Average BW is 3,265 grams (σ = 456 g) for female infants and 3,392 grams (σ = 477 grams) for male infants. Among female infants, 4.44% were LBW and 2.52% TLBW. These figures were slightly lower among male infants: 3.16% and 1.38% respectively for LBW and TLBW. Gestational age was similar for female and male infants: 39.4 weeks (σ = 1.1 weeks).

Table 2 describes the mean (Standard Deviation) estimated exposures to NO_2_ and PM_10_ by pregnancy period. Variations between the windows of exposure are very slight: on average, exposure levels during pregnancy vary between 52.4 and 53 μg/m^3^ for NO_2_ and between 29.5 and 30.1 μg/m^3^ for PM_10_.

**Table 2:** Mean concentrations of NO_2_ and PM_10_ (in μg/m^3^) and standard deviations during four windows of exposure: Entire pregnancy and first (T1), second (T2) and third (T3) trimesters of pregnancy.

	Mean	Standard deviation
NO_2_ (μg/m^3^)		
Entire pregnancy	52.7	7,8
T1	53.0	10,5
T2	52.8	10,8
T3	52.4	10,8
PM_10_ (μg/m3)		
Entire pregnancy	29.9	2,5
T1	30,1	4,3
T2	29,8	4,5
T3	29,5	4,4

### Adverse birth outcomes related to air pollution

Tables 3 and 4 shows the measures of association for a 10 μg/m^3^ increase in air pollutants by trimester of pregnancy from the regression models (linear and logistic regression to model BW and LBW-TLBW, respectively), adjusted for individual confounders (for Female infants Table 3- and Male infants Table 4-, separately). There is a clear infant sex difference in terms of the magnitude of associations (βs and ORs), p-values, critical windows of exposure (T1, T2, T3 and entire pregnancy) and outcomes (BW, LBW and TLBW).

**Table 3:** Measures of associations and p-value (or 95% confidence interval) for 10μg/m^3^ increase in concentrations of air pollutants by trimesters of pregnancy from regression models (defined as model 1 in §Material and methods) adjusted for individual covariates for girls.

		GIRLS					
		Birth weight		Low birth weight		Term low birth weight	
Air pollutants	Window of exposure	β	p-value	OR	[CI95%]	OR	[CI95%]
NO_2_	Entire pregnancy	-3.2	0.157	1.02	[0.98;1.035]	1	[0.99;1.007]
	T1	-0.1	0.962	1	[0.97;1.025]	1	[0.99;1.003]
	T2	***-4*.*8***	***0*.*019***^1^	1.02	[0.99;.046]	1.02	[0.98;1.05]
	T3	2.7	0.132	0.99	[0.96;1.01]	0.96	[0.95;1.01]
PM_10_	Entire pregnancy	-9.3	0.190	1.01	[0.92;1.10]	0.99	[0.97;1.02]
	T1	-1.4	0.746	1	[0.98;1.01]	1.02	[0.94;1.08]
	T2	***-8*.*8***	***0*.*028***^2^	1.005	[0.99;1.02]	1.00	[0.98;1.01]
	T3	1.1	0.799	0.99	[0.94;1.04]	0.96	[0.94;1.08]

Adjusted on: maternal age, gestational age, woman’s primary maternity (yes or no), and maternity classification (class I: low risk, class II: moderate risk and class III: high risk).

^1^: Controlling the False discovery rate (FDR) at α = 0.05 following the method of Benjamini & Hochberg [18], FDR α-risk = 0.001;

^**2**^: FDR α-risk = 0.003.

Bold character presents significant result at 5%; Bold and italic character presents significant result at FDR-risk; Ref: referent value. β corresponds to regression coefficient of a linear regression; OR: Odds Ratio. NO_2_: Nitrogen dioxide. PM_10_: Particulate matter < 10 μm in aerodynamic diameter.

**Table 4:** Measures of associations and p-value (or 95% confidence interval) for 10μg/m^3^ increase in concentrations of air pollutants by trimesters of pregnancy from regression models (defined as model 1 in §Material and methods) adjusted for individual covariates for boys.

		BOYS					
		Birth weight		Low birth weight		Term low birth weight	
Air pollutants	Window of exposure	β	p-value	OR	[CI95%]	OR	[CI95%]
NO_2_	Entire pregnancy	-2.6	0.261	1.07	[0.99;1.015]	1.05	[0.97;1.17]
	T1	0.2	0.933	1.05	[0.98;1.13]	1.05	[0.97;1.14]
	T2	-2.6	0.212	0.97	[0.89;1.04]	0.93	[0.85;1.02]
	T3	0.3	0.859	**1.07**	**[1.00;1.14]**^3^	**1.10**	**[1.02;1.19]**^5^
PM_10_	Entire pregnancy	***-15*.*4***	***0*.*034***^1^	1.23	[0.99;1.6]	1.24	[0.93;1.72]
	T1	***-9*.*2***	***0*.*040***^2^	1.15	[0.99;1.37]	1.11	[0.91;1.36]
	T2	-2.7	0.504	0.98	[0.85;1.14]	1.0	[0.83;1.20]
	T3	-5.4	0.220	**1.17**	**[1.01;1.32]** ^4^	**1.19**	**[1.01;1.39]**^6^

Adjusted on: maternal age, gestational age, woman’s primary maternity (yes or no), and maternity classification (class I: low risk, class II: moderate risk and class III: high risk).

^1^: Controlling the False discovery rate (FDR) at α = 0.05 following the method of Benjamini & Hochberg [18], FDR α-risk = 0.007;

^**2**^: FDR α-risk = 0.009;

^**3**^FDR α-risk = 0.01;

^**4**^FDR α-risk = 0.006;

^**5**^FDR α-risk = 0.001;

^**6**^FDR α-risk = 0.01 [18].

Bold character presents significant result at 5%. Bold and italic character presents significant result at FDR-risk; Ref: referent value. β corresponds to regression coefficient of a linear regression; OR: Odds Ratio. NO_2_: Nitrogen dioxide. PM_10_: Particulate matter < 10 μm in aerodynamic diameter.

#### Birth weight

Among female infants, the only two significant findings are the reduction of BW by 4.8 grams (p-value<0.019) and 8.8 grams (p-value<0.028) during the second trimester (T2) for a 10 μg/m^3^ increase in NO_2_ and PM_10_, respectively. Among male infants, significant reductions in BW were found for different windows of exposure and for PM_10_ only, compared to female infants. BW is reduced by 15.4 grams (p-value<0.034) and 9.2 grams (p-value<0.040) over the entire pregnancy and the first trimester, respectively, for a 10 μg/m^3^ increase of PM_10_.

#### Low birth weight and term low birth weight

There is no significant association among female infants. Among male infants, NO_2_ and PM_10_ exposure estimates during the third trimester of pregnancy were associated with LBW and TLBW: for LBW, the OR was 1.07 CI95%[1.0;1.14] for NO_2_ and equal to 1.17 CI95%[1.01;1.32] for PM_10_; for TLBW, the corresponding figures are: OR = 1.10; CI95%[1.02;1.19] and OR = 1.19; CI95% [1.01;1.39] for NO_2_ and PM_10_, respectively.

### Adverse birth outcomes related to air pollution, taking neighborhood socioeconomic level into account

#### Inter-census versus intra-census block variance

Adjusted for individual variables, between-census-block variation contributed to about 2.5% and 3.6% of total variance in BW among female infants and male infants, respectively. The low ICC values for BW suggest much greater heterogeneity within census tracts (i.e. between individuals) than between census tracts (Table 5). When taking into account the fixed effect of neighborhood socioeconomic status (at Level 2) in the individual-adjusted model (Model 1), between-census-block variation decreased slightly to about 2% and 3% of total variance, respectively. This means that neighborhood socioeconomic status can explain only a small part of between-census-block variation.

**Table 5:** Measure of census blocks variation.

	***GIRLS***		
	**Birth weight**	**Low birth weight**	**Term low birth weight**
	ICC*	MOR **	MOR **
NO2 *Entire pregnancy*	2.1% (2.5%)	1.13 (1.13)	1.08 (1.1)
NO2 (by trimester)	2.3% (2.5%)	1.13 (1.14)	1.09 (1.1)
PM10 *Entire pregnancy*	2.3% (2.5%)	1.13 (1.13)	1.08 (1.09)
PM10 (by trimester)	2.3% (2.5%)	1.13 (1.13)	1.08 (1.09)
	***Boys***		
	ICC*	MOR **	MOR **
NO2 *Entire pregnancy*	3.1% (3.6%)	1.1 (1.19)	1.01 (1.16)
NO2 (by trimester)	3.2% (3.6%)	1.14 (1.19)	1.05 (1.16)
PM10 *Entire pregnancy*	3.2% (3.6%)	1.14 (1.19)	1.07 (1.16)
PM10 (by trimester)	3.1% (3.6%)	1.13 (1.19)	1.13 (1.16)

ICC: Intraclass Coefficient Correlation. MOR: Median Odds Ratio.

*: in parenthesis, the ICC value before considering the class of neighborhood socioeconomic level.

**: in parenthesis, the MOR value before considering the class of neighborhood socioeconomic level.

Model 2 estimated MOR values to vary between 1.1 and 1.14 for the risk of LBW and between 1.01 and 1.13 for the risk of TLBW. By comparison with the measure of association between LBW among female infants and air pollution, the MOR value, equal to 1.13, means that the unexplained variability of risks between census blocks is higher than can be explained by air pollution alone. Among male infants, when analyzing the risk of TLBW, for instance, the inverse is found: the MOR value of close to 1 is lower than the measure of association of the combined effect of air pollution and neighborhood socioeconomic status, showing both that air pollution does play a role, and that this role is influenced by neighborhood socioeconomic profile.

#### Relationship between birth outcome and air pollution exposure plus neighborhood socioeconomic level

In this section we describe *i)* the association between different birth outcomes where air pollution exposure was measured at various windows of exposure and neighborhood socioeconomic level was measured at census level, *ii)* potential interaction between air pollution and socioeconomic level and *iii)* the effect of air pollution according to socioeconomic level.

**Fig 1:**
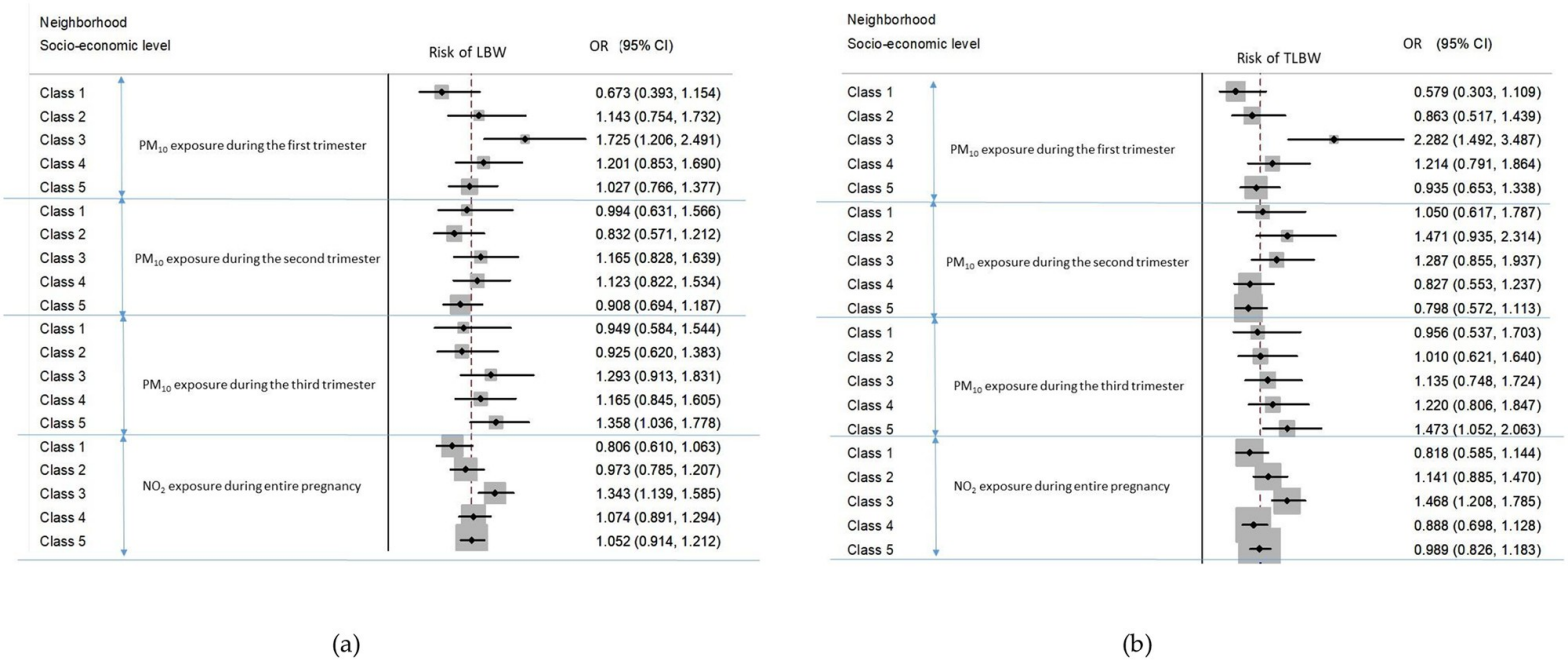
Effect of air pollution according neighborhood socio-economic category: (a) risk of LBW; (b) risk of TLBW. These results were presented because the interaction term between air pollution and socioeconomic categories were statistically significant.

Tables 6 and 7 shows measures of association for a 10 μg/m^3^ increase in air pollutants by trimester of pregnancy, using multilevel regression models adjusted for individual confounders and taking into account neighborhood socioeconomic level as either a confounder or an effect modifier. Analysis of the effect of air pollution exposure according to socioeconomic level was presented in Fig 1.

**Table 6:** Association between adverse birth outcome (BW, LBW, TLBW) and air pollution exposure during pregnancy plus neighborhood socioeconomic level for girls using multilevel model (defined as Model 2 in §Material and methods).

			GIRLS					
			Birth weight		Low birth weight		Term low birth weight	
Window of exposure	Variables	Spatial Level	β	p-value	OR	[CI95%]	OR	[CI95%]
**Entire pregnancy**								
	NO_2_	Level-1 individuals	-3.3	0.149	1.03	[0.98; 1.01]	0.99	[0.98; 1.01]
	**Socio-economic level**:	Level-2 Census blocks						
	Class 1		Ref	---	Ref	---	Ref	---
	Class 2		11.9	0.066	1	[0.82; 1.23]	1.02	[0.82; 1.27]
	Class 3		1.34	0.833	0.99	[0.82; 1.21]	0.97	[0.77; 1.21]
	Class 4		**12.3**	**0.048**	1.03	[0.85; 1.25]	1.02	[0.83; 1.27]
	Class 5		-2.47	0.688	1.14	[0.95; 1.37]	1.10	[0.9; 1.36]
**Exposure by trimester**								
	***NO***_***2***_	Level-1 individuals						
	T1		<10–3	0.967	1	[0.95; 1.06]	0.97	[0.91; 1.03]
	T2		**-4.7**	**0.021**^1^	1.04	[0.98; 1.11]	1.04	[0.97; 1.12]
	T3		2.6	0.166	0.97	[0.91; 1.02]	0.96	[0.91; 1.03]
	***Socio-economic level***	Level-2 Census blocks						
	Class 1		Ref	---	Ref	---	Ref	---
	Class 2		11.8	0.069	1	[0.82; 1.22]	1.02	[0.82; 1.28]
	Class 3		1.2	0.852	0.99	[0.82; 1.21]	0.97	[0.77; 1.21]
	Class 4		**12.3**	**0.048**	1.03	[0.85; 1.25]	1.02	[0.82; 1.27]
	Class 5		-2.4	0.696	1.14	[0.94; 1.37]	1.10	[0.9; 1.36]
**Entire pregnancy**								
	***PM***_***10***_	Level-1 individuals	-9.4	0.188	1.03	[0.84;1.27]	1.0	[0.78; 1.25]
	***Socio-economic level***	Level-2 Census blocks						
	Class 1		Ref	---	Ref	---	Ref	---
	Class 2		11.8	0.068	1	[0.82; 1.22]	1.02	[0.81; 1.27]
	Class 3		1.24	0.845	0.99	[0.82; 1.21]	0.97	[0.77; 1.21]
	Class 4		**12.5**	**0.045**	1.03	[0.85; 1.24]	1.02	[0.83; 1.27]
	Class 5		-2.4	0.701	1.13	[0.94; 1.37]	1.10	[0.89; 1.36]
**Exposure by trimester**								
	***PM***_***10***_	Level-1 individuals						
	T1		-1.4	0.748	1	[0.87; 1.15]	1.03	[0.88; 1.19]
	T2		**-8.7**	**0.031**^2^	1.05	[9.93; 1.19]	1.0	[0.87; 1.15]
	T3		0.6	0.893	0.98	[0.86; 1.11]	0.96	[0.83; 1.11]
Level-2 Census blocks	***Socio-economic level***							
	Class 1		Ref	---	Ref	---	Ref	---
	Class 2		11.7	0.070	1	[0.82; 1.23]	1.02	[0.82; 1.27]
	Class 3		1.2	0.853	0.99	[0.82; 1.21]	0.97	[0.77; 1.21]
	Class 4		**12.4**	**0.047**	1.03	[0.85; 1.24]	1.02	[0.82; 1.27]
	Class 5		-2.4	0.697	1.14	[0.94; 1.37]	1.10	[0.89; 1.36]

This table describe measures of association and p-values for 10μg/m^3^ increases of air pollutants in trimesters of pregnancy from multilevel models (defined as Model 2 in §Material and methods) adjusted for all individual covariates. Here, model include only the main effect of air pollution and the main effect of the socioeconomic level on the adverse birth outcome. No modifier effect has been considered.

Adjusted on: gestational age, mother’s age, woman’s primary maternity (yes or no), and maternity classification (class I: low risk, class II: moderate risk and class III: high risk).

Bold character presents significant result at 5%. Ref: referent value. β corresponds to regression coefficient of a linear regression; OR: Odds Ratio. “---”not applicable.

SES: Neighborhood socioeconomic status With Class 1 corresponding to the less deprived and class 5 the most deprived; NO_2_: Nitrogen dioxide. PM_10_: Particulate matter < 10 μm in aerodynamic diameter.

*significant interaction for Low birth weight and Term Low birth weight; ** significant interaction only for **Term Low birth weight**.

^1^: Controlling the False discovery rate (FDR) at α = 0.05 following the method of Benjamini & Hochberg [18], FDR α-risk = 0.002;

^**2**^: FDR α-risk = 0.004.

**Table 7:** Association between adverse birth outcome (BW, LBW, TLBW) and air pollution exposure during pregnancy plus neighborhood socioeconomic level for boys using multilevel model (defined as Model 2 in §Material and methods).

			BOYS					
Window of exposure	Variables	Spatial level	Birth weight		Low birth weight		Term low birth weight	
**Entire pregnancy**			β	p-value	OR	[CI95%]	OR	[CI95%]
	**NO**_**2**_	Level-1 individuals	-3.8	0.111	0.97	[0.61; 1.05]	0.82	[0.59; 1.14]
	***Socio-economic level***	Level-2 Census blocks						
	Class 1		Ref	---	Ref	---	Ref	---
	Class 2		1.9	0.772	0.97	[0.76; 1.24]	0.88	[0.66; 1.19]
	Class 3*		-9.5	0.150	1.03	[0.82; 1.31]	0.92	[0.68; 1.23]
	Class 4		**-12.7**	**0.050**	1.03	[0.82; 1.30]	0.90	[0.68; 1.29]
	Class 5		**-24.9**	**<0.001**	**1.34**	**[1.08; 1.67]**	1.20	[0.92; 1.57]
**Exposure by trimester**								
	**NO**_**2**_	Level-1 individuals						
	T1		-0.2	0.915	1.05	[0.98; 1.13]	0.89	[0.70; 1.14]
	T2		-2.9	0.162	0.96	[0.89; 1.04]	0.93	[0.85; 1.02]
	T3		-0.2	0.930	**1.07**	**[1.003; 1.15]**^3^	**1.1**	**[1.015; 1.19]**^5^
	***Socio-economic level***	Level-2 Census blocks						
	Class 1		Ref	---	Ref	---	Ref	---
	Class 2		1.9	0.775	0.97	[0.76; 1.24]	0.89	[0.66; 1.19]
	Class 3**		-9.5	0.149	1.06	[0.82; 1.32]	0.91	[0.68; 1.22]
	Class 4		*-12*.*6*	*0*.*051*	1.03	[0.83; 1.31]	0.92	[0.69; 1.21]
	Class 5		**-24.9**	**<0.001**	**1.34**	**[1.08; 1.68]**	1.19	[0.92; 1.56]
**Entire pregnancy**								
	**PM**_**10**_	Level-1 individuals	**-18.5**	**0.012**^1^	1.24	[0.99; 1.61]	1.25	[0.94; 1.75]
	***Socio-economic level***	Level-2 Census blocks						
	Class 1		Ref	---	Ref	---	Ref	---
	Class 2		1.8	0.788	0.96	[0.75; 1.23]	0.88	[0.66; 1.18]
	Class 3		-9.5	0.148	1.06	[0.84; 1.35]	0.97	[0.74; 1.29]
	Class 4		**-12.7**	**0.049**	1.02	[0.81; 1.29]	0.91	[0.69; 1.2]
	Class 5		**-25.1**	**<0.001**	**1.33**	**[1.07; 1.67]**	1.19	[0.91; 1.56]
**Exposure by trimester**								
	PM_10_	Level-1 individuals						
	T1		**-10.2**	**0.025**^2^	0.79	[0.48; 1.25]	0.65	[0.37; 1.17]
	T2		-3.5	0.392	0.99	[0.85; 1.15]	1.01	[0.84; 1.21]
	T3		-6.9	0.119	**1.17**	**[1.02; 1.38]**^4^	1.19	[0.999; 1.45]
	***Socio-economic level***	Level-2 Census blocks						
	Class 1		Ref	---	Ref	---	Ref	---
	Class 2		1.8	0.780	0.97	[0.76; 1.25]	0.9	[0.67; 1.22]
	Class 3*		-9.5	0.151	1.06	[0.83; 1.34]	0.93	[0.69; 1.24]
	Class 4		**-12.7**	**0.048**	1.04	[0.83; 1.31]	0.93	[0.70; 1.23]
	Class 5		**-25.1**	**<0.001**	**1.35**	**[1.08; 1.69]**	1.21	[0.93; 1.58]

This table describe measures of association and p-values for 10μg/m^3^ increases of air pollutants in trimesters of pregnancy from multilevel models (defined as Model 2 in §Material and methods) adjusted for all individual covariates. Here, model include only the main effect of air pollution and the main effect of the socioeconomic level on the adverse birth outcome. No modifier effect has been considered.

Adjusted on: gestational age, mother’s age, woman’s primary maternity (yes or no), and maternity classification (class I: low risk, class II: moderate risk and class III: high risk).

Bold character presents significant result at 5%. Ref: referent value. β corresponds to regression coefficient of a linear regression; OR: Odds Ratio. “---”not applicable.

SES: Neighborhood socioeconomic status With Class 1 corresponding to the less deprived and class 5 the most deprived; NO_2_: Nitrogen dioxide. PM_10_: Particulate matter < 10 μm in aerodynamic diameter.

*significant interaction for **Low birth weight and Term Low birth weight**;

** significant interaction only for **Term Low birth weight**.

^1^: Controlling the False discovery rate (FDR) at α = 0.05 following the method of Benjamini & Hochberg [18], FDR α-risk = 0.002;

^**2**^: FDR α-risk = 0.004;

^**3**^: FDR α- risk = 0.008;

^**4**^: FDR α- risk = 0.005;

^**5**^: FDR α- risk = 0.003.

#### Birth weight

*Effect of air pollution and socioeconomic level*. With regard to the effects of air pollution exposure, our results revealed that for female infants, beta coefficients are -4.7 (p-value<0.021) and -8.7 grams (p-value<0.031) during the second trimester for a 10 μg/m^3^ increase in NO_2_ and PM_10_, respectively. For male infants, BW is lower for a 10 μg/m^3^ increase in PM_10_ only.

Analyzing the effect of socioeconomic neighborhood level, we found that male infants living in the most deprived census blocks (Class 4 and Class 5) always show reduced BW compared to those living in the least deprived census blocks (Class 1)—a reduction which ranges from -12.6 grams (p-value<0.051) to -25.1 grams (p-value<0.001) across census blocks.

*Interaction air pollution and socioeconomic level*. As shown in Tables 6 and 7, no significant interaction was found between air pollution and neighborhood socioeconomic level among either female or male infants.

#### Low birth weight and term low birth weight

*Effect of air pollution and socioeconomic level*. Among female infants, we found no significant association with either air pollution or neighborhood socioeconomic status. By contrast, several associations were found among male infants. In terms of windows of exposure, exposure to NO_2_ or PM_10_ during the third trimester significantly increases the risk of LBW and TLBW, whereas this effect was significant throughout the entire pregnancy period for PM_10_ exposure only. Regarding the main effect of neighborhood socioeconomic level, an increased risk of both LBW and TLBW was found for infants living in the most deprived census blocks (Class 5) compared to the least deprived (Class 1).

*Interaction air pollution and socioeconomic level*. The interaction between air pollution and census block deprivation characteristics is less clear: the interaction term was found to be statistically significant for air pollution estimates only in Class 3 census blocks, either throughout the pregnancy or during the first trimester.

*Effect of air pollution according to socioeconomic level*. As shown in Fig 1, we found a significant modification effect at neighborhood socioeconomic level among male infants only; those living in Class 3 census blocks being more at risk.

#### Term BW and preterm birth

We used gestational age to investigate the risk of preterm birth as well as BW at term associated with air pollution exposure. The results have been summarized as supplementary tables (see S1 Appendix).

#### Sensitivity analysis

We performed sensitivity analysis using the spline function to analyze maternal age as a continuous variable. Using the AIC statistic, we compared the fit of three different models where the maternal age variable is included as (1) continuous, (2) 5 classes and (3) spline function.

These additional analyses revealed that, where age is included as a categorical variable (5 maternal age classes), the quality of the fit is broadly improved, compared to the model that includes the maternal age variable as a continuous variable with the spline function, since here, the AICI is only slightly improved (data not shown).

As with maternal age, we performed sensitivity analysis using the spline function to include the socioeconomic index as a continuous variable. Using the AIC statistic, we compared the fit of three different models where the socioeconomic index variable is included as (1) continuous, (2) quintile and (3) spline function (data not shown).

These additional analyses revealed that while including the socioeconomic index as a quintile variable broadly improved fit compared to the model that includes the socioeconomic variable as a continuous variable, with the spline function the AICI is only slightly improved (data not shown).

## Discussion

### Air pollution health effects

Our study revealed significant associations between BW and air pollution that were coherent with those described by other studies. BW overall decreased, and the risk of LBW and TLBW was increased with a 10 μg/m^3^ increase in exposure to air pollution during pregnancy, for both NO_2_ and PM_10_. A recent study [19] conducted in Norway found a comparable magnitude of BW decreases associated with NO_2_ throughout the pregnancy, though this is not statistically significant (7.4 grams decrease on average). In London in 2017, Smith et al. [20] found an OR for TLBW equal to 1.03 (CI_95%_ = [1.0;1.06]) for an IQR increase of NO_2_ (IQR = 8.6 μg/m^3^) and to 1.03 (CI_95%_ = [0.99;1.07]) for PM10 (IQR = 3 μg/m^3^). In Spain [21], Arroyo et al. found a global relative risk for each province and autonomous community of LBW equal to 1.104 (CI_95%_ = [1.072;1.138]) and to 1.091 (CI_95%_ = [1.059;1.124]) for 10 μg/m^3^ increases in PM_10_ and NO_2_, respectively. In China [22], comparable measures of association were also found: the OR of LBW was 1.018 (95% CI 1.01–1.04) for each 10 μg/m^3^ increment in PM_10_ throughout the pregnancy. The authors also demonstrated that exposure during both the early and late stages of pregnancy led to higher risk of preterm birth and LBW related to PM_2.5_, PM_10_, NO_2_, SO_2_, CO, O_3_. They concluded that preventive actions aimed at reducing or avoiding exposure were particularly important for pregnant women. However, like most studies, they ignored the possible effect of modification by infant sex, and adjusted on infant sex in their statistical analysis to take into account the well-known weight difference at birth between male and female infants.

### Infant sex difference air pollution effects

Our study found also infant sex difference: while no significant association was revealed among female infants for the risk of LBW or TLBW, these two untoward birth outcomes are more likely among male infants when exposure to either NO_2_ or PM_10_ increases during the third trimester of pregnancy. A meta-analysis published in 2007 showed that the difference in the risk of LBW between the two infant sexes suggests an interaction; however, too few studies had stratified the analysis, so that it was impossible to reach a formal conclusion. In a large UK cohort [23] that used an innovative exposure model, the author suggests results are in favor of a differential infant sex effect of air pollution throughout the pregnancy; however, female infants appeared more vulnerable—although the difference was not statistically significant.

Results from US studies were contrasted. Whereas Bell et al. concluded that the relationship between air pollution and LBW did not differ by infant sex [1], Rhee et al., found that the reduction of birthweight related to PM_2.5_ was more pronounced in male infants [24]. In addition, Lakshman et al. revealed that higher PM_2.5_ and lower BW was significant in the male infants of obese mothers [25].

Although a more frequently stated hypothesis is that male infants are more vulnerable than female infants, a recent study suggests that infant sex vulnerability might vary throughout the gestation period [26]. More precisely, Erickson et al. explain that in the first few weeks of pregnancy, due to a higher proportion of anomalies in male embryos, male losses are higher, whereas later on in the first trimester and during the second, there are increased losses of female fetuses. From mid-gestation onward, there is a higher rate of male fetal mortality. This is consistent with the critical window of vulnerability identified in our study for male infants only, as we found that higher exposure to air pollution during the third trimester significantly increases the risk of adverse birth outcomes among male infants. The risk estimate for female infants during the first trimester of exposure were not significant.

### Neighborhood socioeconomic level modification

In our study, neighborhood socioeconomic level was significantly correlated with BW, either as a lone risk factor (especially among female infants) or as an effect modifier (among male infants). The significant interaction we found among male infants supports the vulnerable hypothesis, while neighborhood socioeconomic level appears as an additional risk factor, independent of air pollution effect among female infants. There is substantial evidence for adverse consequences of neighborhood socioeconomic level on birth outcomes, irrespective of infant sex, even after adjusting on individual socioeconomic status [11, 12, 27]. Few studies have investigated possible differential associations between air pollution and birth outcomes across neighborhood socioeconomic characteristics. A majority of studies have found a stronger association between air pollution and birth outcomes in more deprived socioeconomic areas [28–30]. In 2010, Morello-Frosch et al. [29] showed a greater reduction in BW with exposure to NO_2_ among infants born to mothers residing in more deprived census tracts (where more than 22% of the population are living in poverty) than those in less deprived census blocks (with less than 22% of the population living in poverty). BW reduction was 13 grams versus 6 to 9 grams, respectively. Conversely, in 2008 Généreux et al. [31] demonstrated that living closer to a highway increased the risk of LBW only when mothers resided in the most socioeconomically privileged areas. We do not know whether this finding is consistent with our results, which suggest that exposure to NO_2_ or PM_10_ during the first trimester elicits more acute effects among male infants born in the middle categories of the neighborhood socioeconomic index. Further investigation is needed to better understand whether the discrepancies in these findings can be put down to the specificities of each study area.

### Sensitivity analysis on individual socioeconomic variables

Regarding individual socioeconomic variables (maternal education and occupational status of the parent), about a third of the values were missing. It is important to note that the missing values are not randomly distributed across the study area. There is an association between the rate of individual missing values and neighborhood socioeconomic level: individual missing values are twice as high in the ten most deprived census blocks, compared to the ten most privileged. Using census data collected in the city of Paris by the National Institute of Statistics and Economic (INSEE—2012), we randomly attributed an education level and an occupational status to mothers and fathers with missing values in order to obtain comparable individual and neighborhood distributions in terms of education level and occupational status. The same statistical analysis was performed (Models 0, 1 and 2) and similar associations were found.

### Strengths and limitations

The main strengths of our study are large sample size (more than 100,000 births), adjustment for several known individual confounding factors, and the exposure assessment method, which combines fine spatial scale estimates with temporal variations of air pollution within census blocks.

While this spatial and temporal accuracy tends to reduce exposure misclassification, our study (like most epidemiological studies dealing with exposure to air pollution) ignored women’s mobility during pregnancy. Because no data were available on personal mobility and time-activity patterns, our exposure estimates were based on maternal residential census block at time of delivery. In a recent study, we assessed the impact of occupational mobility among pregnant women living in Paris on their exposure estimates to NO_2_ [32] and demonstrated that occupational mobility had a low impact on exposure levels, while taking into account that the commuting mode had a greater impact, particularly among women living in the most deprived census blocks.

A further weakness is air pollution exposure assessment (including lack of consideration of space-time activity or indoor air levels) which may in turn result in exposure misclassification. In our study, we used air pollution exposure based on modeling resulting from a dispersion model, then weighted by daily variation (measured at census block level) using concentrations from monitoring stations. However, our air exposure estimate did not take into account other determinants/input parameters, such as the time spent in different places as the house, public transport usage and workplace and indoor air level exposure. Due to lack of data, it was not possible to consider these parameters in our study. However, some studies have reported that considering daily activity across a territory would have only slightly modified the estimated outdoor exposure to PM_2.5_ and NO_2_.

This study was based on data obtained from the first medical certificate, which has a very high rate of completeness (93% of the total birth) in Paris [13].

However, though this document provided data for several risk factors, other important known risk factors for birth outcomes exist, which were either unavailable from the information system (maternal BMI, for instance) or had a high number of missing values (maternal smoking and alcohol consumption status during pregnancy). For instance, information about smoking or alcohol consumption during pregnancy is available on the medical certificate. However, almost all the women’s answers were “no”, and the other part of the answer was missing. One option was to formulate the hypothesis that all the missing values were “yes”; however, we considered this a crude hypothesis and preferred not to include these variables in the model. Therefore, consideration of these factors was not possible in our study, and this may have affected both our risk estimates and the variance explained by census block level.

Due to the absence of these individual variables, it is difficult to understand exactly what is captured in the intra-census block variance. We cannot exclude the possibility that part of the intra-variance could be explained by the absence of individual variables, while another part could be more related to neighborhood characteristics.

Lastly, we know that geographical level is an important consideration in such a study, independent of the statistical method implemented. The size of the spatial unit has to be as small as possible to maximize the homogeneity of variables within each area, as well as major differences between areas. In our study, we used socioeconomic data collected at the smallest French census block level (grouping on average 2000 inhabitants). Census blocks are defined so as to maximize uniformity in terms of population size, socioeconomic and demographic characteristics, land use, and zoning, thus reducing the risk of ecological bias.

### Public health implications

Our findings suggest the existence of a differential association between air pollution and BW according to both neighborhood socioeconomic level and infant sex. However, due to multiple statistical tests and controlling the FDR at α = 0.05 (following the method of Benjamini & Hochberg [18]), all significant associations became either not statistically significant or borderline. Additional research—in the form of both experimental and epidemiological studies—is called for, to confirm (or refute) such findings.

Defining the underlying mechanisms is a challenge: adverse birth outcomes may arise out of a disproportionate exposure burden and/or a differential susceptibility to exposure—again, additional investigation is called for. Our findings on the role of neighborhood socioeconomic level, especially among male infants, reinforce the need to specifically design studies aimed at disentangling which dimensions of the neighborhood socioeconomic pattern differentially increase susceptibility, and elucidating their physiological and behavioral mechanisms.

Overall, the increased risk of air pollution to BW is relatively low compared to other well-known risk factors, yet the total number of pregnant women potentially affected is significant. In addition, identifying the subpopulations that are most vulnerable to the effects of air pollution remains a public health research concern. To support environmental policies aiming to tackle air pollution, quantitative health impact assessments (HIAs) stand out as a decision-making tool, because they provide valuable information regarding the future health effects of a potential plan or policy. To build efficient policies, it is important to establish a complete socioeconomic and health-related assessment at a fine spatial scale. This will allow us to identify those categories of the population that accumulate multiple individual risk factors (in addition to those measured in homes). Very few studies have quantified the health impact of air pollution reduction at a small spatial scale, stratified by neighborhood socioeconomic level and for a specific subpopulation (such as pregnant women). However, these studies are crucial to the development of targeted policies—environmental and social policies in particular. The combination of an HIA with a clustering approach to map health impact by socioeconomic deprivation level may constitute a powerful priority-setting tool, and guide policymakers in their choice of environmental policies.

## Supporting information

S1 Appendix (DOCX)
