# Supplementary material for: Are the effects of air pollution on birth weight modified by infant sex and neighborhood socioeconomic deprivation? A multilevel analysis in Paris (France)
Source: PLoS One. 2021 Apr 15;16(4):e0247699. doi: 10.1371/journal.pone.0247699 (PMC8049242; doi:10.1371/journal.pone.0247699)
Supplement: S1 Appendix — (DOCX) [file pone.0247699.s001.docx]

|  |  | | **GILRS** | | | | |  |  |  |
| --- | --- | --- | --- | --- | --- | --- | --- | --- | --- | --- |
|  |  | | Term birth weight | | | Preterm birth | |  |  |  |
| Air pollutants | Window of exposure | | β | | p-value | OR | [CI95%] |  |  |  |
| NO_2_ | Entire pregnancy | | -0.8 | | 0.74 | 1.02 | [0.99 ;1.05] |  |  |  |
|  | T1 | | -0.3 | | 0.89 | 1.00 | [0.97 ;1.02] |  |  |  |
|  | T2 | | 2.2 | | 0.32 | 0.99 | [0.97 ;1.02] |  |  |  |
|  | T3 | | -3.1 | | 0.12 | **1.03** | **[1.01 ;1.05]** |  |  |  |
| PM_10_ | Entire pregnancy | | 8.6 | | 0.27 | 0.95 | [0.87;1.03] |  |  |  |
|  | T1 | | 2.9 | | 0.54 | 0.95 | [0.91;1.01] |  |  |  |
|  | T2 | | 5.6 | | 0.21 | 0.96 | [0.92;1.01] |  |  |  |
|  | T3 | | -0.06 | | 0.99 | 1.02 | [0.97;1.07] |  |  |  |
|  | |  | |  | | | | | |  |

| Adjusted on: maternal age, woman’s primary maternity (yes or no), and maternity classification (class I: low risk, class II: moderate risk and class III: high risk). |
| --- |
| Legend: Bold character presents significant result at 5%. Ref: referent value. β corresponds to regression coefficient of a linear regression; OR: Odds Ratio. NO_2_: Nitrogen dioxide. PM_10_: Particulate matter < 10 μm in aerodynamic diameter. |

|  |  | **BOYS** | | | |
| --- | --- | --- | --- | --- | --- |
|  |  | Term birth weight | | Preterm birth | |
| Air pollutants | Window of exposure | β | p-value | OR | [CI95%] |
| NO_2_ | Entire pregnancy | 0.4 | 0.87 | 1.01 | [0.95;1.07] |
|  | T1 | 1.3 | 0.55 | 0.97 | [0.93 ;1.02] |
|  | T2 | 3.7 | 0.12 | 0.98 | [0.93 ;1.04] |
|  | T3 | **-4.9** | **0.02** | **1.06** | **[1.01 ;1.22]** |
| PM_10_ | Entire pregnancy | 3.3 | 0.69 | 0.91 | [0.76;1.09] |
|  | T1 | **-9.1** | **0.043** | **0.85** | **[0.76;0.95]** |
|  | T2 | -2.9 | 0.48 | 0.99 | [0.89;1.09] |
|  | T3 | -5.7 | 0.19 | 1 | [0.90;1.12] |

| Adjusted on: maternal age, woman’s primary maternity (yes or no), and maternity classification (class I: low risk, class II: moderate risk and class III: high risk). |
| --- |
| Legend: Bold character presents significant result at 5%. Ref: referent value. β corresponds to regression coefficient of a linear regression; OR: Odds Ratio. NO_2_: Nitrogen dioxide. PM_10_: Particulate matter < 10 μm in aerodynamic diameter. |

|  |  |  | **GIRLS** | | | |
| --- | --- | --- | --- | --- | --- | --- |
|  |  |  | Term birth weight | | Preterm birth | |
|  |  | Window of exposure | β | P value | OR | [CI95%] |
| Level-1 individuals | NO_2_ | Entire pregnancy | 0.8 | 0.75 | **1.06** | **[1.00 ;1.13]** |
| Level-2 Census blocks | SES | Class 1 | ref | --- | ref | --- |
|  |  | Class 2 | 6.8 | 0.33 | 1.05 | [0.86 ;1.26] |
|  |  | Class 3 | -2.5 | 0.71 | **1.26** | **[1.05 ;1.51]** |
|  |  | Class 4 | 8.6 | 0.20 | **1.25** | **[1.05 ;1.49]** |
|  |  | Class 5 | -7.6 | 0.25 | **1.61** | **[1.36 ;1.90]** |
| Level-1 individuals | NO_2_ | T1 | -0.2 | 0.92 | 1.004 | [0,95 ;1.05] |
|  |  | T2 | 2.2 | 0.32 | 0.99 | [0.94 ;1.05] |
|  |  | T3 | -3.2 | 0.11 | **1.07** | **[1.02 ;1.05]** |
| Level-2 Census blocks | SES | Class 1 | Ref | --- | Ref | --- |
|  |  | Class 2 | 6.9 | 0.32 | 1.04 | [0.87 ;1.26] |
|  |  | Class 3 | -2.4 | 0.72 | **1.26** | **[1.05 ;1.51]** |
|  |  | Class 4 | 8.7 | 0.20 | **1.25** | **[1.05 ;1.49]** |
|  |  | Class 5 | -7.6 | 0.25 | **1.61** | **1.37 ;1.91]** |
| Level-1 individuals | PM_10_ | Entire pregnancy | 8.6 | 0.27 | 0.91 | [0.75 ;1.10] |
| Level-2 Census blocks | SES | Class 1 | Ref | --- | Ref | --- |
|  |  | Class 2 | 6.8 | 0.33 | 1.05 | [0.87 ;1.26] |
|  |  | Class 3 | -2.6 | 0.71 | **1.27** | **[1.06 ;1.52]** |
|  |  | Class 4 | 9.0 | 0.18 | **1.24** | **[1.04 ;1.47]** |
|  |  | Class 5 | -7.2 | 0.28 | **1.59** | **[1.35 ;1.89]** |
| Level-1 individuals | PM_10_ | T1 | 3.1 | 0.55 | 0.91 | [0.81 ;1.03] |
|  |  | T2 | 5.6 | 0.20 | 0.93 | [0.83 ;1.04] |
|  |  | T3 | -0.3 | 0.95 | 1.07 | [0.95 ;1.19] |
| Level-2 Census blocks | SES | Class 1 | Ref | --- | Ref | --- |
|  |  | Class 2 | 6.9 | 0.33 | 1.05 | [0.86 ;1.26] |
|  |  | Class 3 | -2.5 | 0.71 | **1.27** | **[1.06 ;1.51]** |
|  |  | Class 4 | 9.1 | 0.17 | **1.24** | **[1.04 ;1.48]** |
|  |  | Class 5 | -7.2 | 0.28 | **1.60** | **[1.35 ;1.89]** |

|  |  |  | **BOYS** | | | | |
| --- | --- | --- | --- | --- | --- | --- | --- |
|  |  |  | Term birth weight | | Preterm birth | |  |
|  |  | Window of exposure | β | P value | OR | [CI95%] |  |
| Level-1 individuals | NO_2_ | Entire pregnancy | -0.7 | 0.78 | 1.02 | [0.97 ;1.08] |  |
| Level-2 Census blocks | SES | Class 1 | ref | --- | ref | --- |  |
|  |  | Class 2 | 7.5 | 0.31 | 1.03 | [0.87 ;1.23] |  |
|  |  | Class 3 | -4.8 | 0.50 | 0.96 | [0.81 ;1.14] |  |
|  |  | Class 4 | -6.3 | 0.37 | 1.17 | [0.99 ;1.37] |  |
|  |  | Class 5 | **-26.6** | **<0.001** | **1.24** | **[1.07 ;1.45]** |  |
| Level-1 individuals | NO_2_ | T1 | 0.8 | 0.71 | 0.98 | [0.93 ;1.03] |  |
|  |  | T2 | 3.4 | 0.14 | 0.99 | [0.94 ;1.04] |  |
|  |  | T3 | **-5.4** | **0.01** | **1.06** | **[1.01 ;1.21]** |  |
| Level-2 Census blocks | SES | Class 1 | Ref | --- | Ref | --- |  |
|  |  | Class 2 | 7.4 | 0.31 | 1.04 | [0.87 ;1.23] |  |
|  |  | Class 3 | -4.8 | 0.49 | 0.96 | [0.81 ;1.14] |  |
|  |  | Class 4 | -6.3 | 0.37 | 1.17 | [0.99 ;1.37] |  |
|  |  | Class 5 | **-26.7** | **<0.001** | **1.25** | **[1.07 ;1.45]** |  |
| Level-1 individuals | PM_10_ | Entire pregnancy | 0.4 | 0.96 | 0.94 | [0.78 ;1.12] |  |
| Level-2 Census blocks | SES | Class 1 | Ref | --- | Ref | --- |  |
|  |  | Class 2 | 7.4 | 0.31 | 1.04 | [0.88 ;1.23] |  |
|  |  | Class 3 | -4.9 | 0.49 | 0.97 | [0.82 ;1.14] |  |
|  |  | Class 4 | -6.1 | 0.38 | 1.16 | [0.99 ;1.36] |  |
|  |  | Class 5 | **-26.5** | **<0.001** | 1.24 | [1.06 ;1.45] |  |
| Level-1 individuals | PM_10_ | T1 | 2.1 | 0.66 | 0.86 | [0.77 ;0.97] |  |
|  |  | T2 | 6.5 | 0.16 | 0.99 | [0.89 ;1.10] |  |
|  |  | T3 | -7.5 | 0.13 | 1.02 | [0.91 ;1.13] |  |
| Level-2 Census blocks | SES | Class 1 | Ref | --- | Ref | --- |  |
|  |  | Class 2 | 7.4 | 0.31 | 1.04 | [0.88 ;1.23] |  |
|  |  | Class 3 | -4.9 | 0.49 | 0.97 | [0.82 ;1.14] |  |
|  |  | Class 4 | -6.2 | 0.38 | 1.16 | [0.99 ;1.36] |  |
|  |  | Class 5 | -26.4 | <0.001 | 1.24 | [1.06 ;1.45] |  |

Adjusted on: mother's age, woman’s primary maternity (yes or no), and maternity classification (class I: low risk, class II: moderate risk and class III: high risk).

Legend: Bold character presents significant result at 5%. Ref: referent value. β corresponds to regression coefficient of a linear regression; OR: Odds Ratio. “---“ not applicable.

SES: Neighborhood socioeconomic status With Class 1 corresponding to the less deprived and class 5 the most deprived; NO_2_: Nitrogen dioxide. PM_10_: Particulate matter < 10 μm in aerodynamic diameter.
